# Vegetative propagation capacity of invasive alligator weed through small stolon fragments under different treatments

**DOI:** 10.1038/srep43826

**Published:** 2017-03-06

**Authors:** Xuemei Peng, Haiyan Li, Yunfei Yang, Heng Zhi, Chengcheng Li, Jian Guo

**Affiliations:** 1 Key Laboratory of Vegetation Ecology, Ministry of Education, Institute of Grassland Science, Northeast Normal University, Changchun 130024, P. R. China; 2 School of Chemistry and Life Sciences, Guizhou Education University, Guiyang 550018, P. R. China

## Abstract

The ability to propagate via small diaspores is crucial for the invasion of a clone plant that does not reproduce sexually in its introduced range. We investigated the effects of node and internode adjacent mode, fragment type, burial orientation and position of the node in relation to the soil surface on the sprouting and growth of alligator weed (*Alternanthera philoxeroides* (Martius) Griseb.). All the factors had effects and interaction effects on the sprouting rate and growth. As a whole fragment in all treatments, the fragments with basal node buried upward on the soil surface, exhibited the best above-ground growth and root growth. The one-node fragment with basal node buried downward above the soil surface and upward under the soil surface significantly decreased the above-ground growth and root growth compared to that of the two-node fragment. Therefore, the one-node fragments were more affected by environmental conditions than the two-node fragments. The results indicated that reducing the number of nodes of a fragment and burying the node under the soil or orienting it downward above the soil surface could be applied to control the invasion of alligator weed.

Biological invasions seriously influence and threaten global biodiversity, species persistence and ecosystem services^1^,^2^. The factor underlying the invasiveness of alien species in their introduced range is a hot topic in invasion biology. An important hypothesis is that invasive plants have strong capabilities for dispersal and reproduction^3^. In plants, dispersal is generally passive, involving the transport of seeds or other diaspores (dispersal units) away from the parent plant by vectors such as animals, wind, and water^4^. The process of reproduction generally includes vegetative propagation and sexual propagation ability. For plants that do not reproduce sexually and whose primary means of dispersal is through diaspores in their introduced range (e.g., alligator weed)^5^,^6^, understanding the capability for dispersal and reproduction via vegetative propagation, especially fragmentation propagation, is crucial to investigating the invasive process and controlling dispersal and invasion. Understanding what determines the survival and growth of small clonal fragments (e.g., stolons, rhizomes or roots) is of both scientific and practical interest.

If clone fragmentation occurs, newly produced offspring ramets cannot benefit from internal support through physiological integration. In stoloniferous species, the short-term storage of reserves (e.g., carbohydrates) in internodes and node bases may enhance the survival of disconnected ramets that do not have leaves to perform photosynthesis and do not have roots to assimilate water and nutrients from soil^7^. Previous studies showed that the newly formed ramets of alligator weed remobilized the resources stored in stolon internodes and nodes, and the survival of ramets increased with the increasing length of the attached internodes^8^. Therefore, fragments with internodes play an important role in the establishment of the population.

After fragments with internodes break off, the adjacent mode of nodes and internodes varies: a node with a basal internode (apical node), a node with an apical internode (basal node), a node with both an apical internode and a basal internode (middle node) or two end nodes with the same middle internode (Fig. 3). When the stolon fragments are attached to the parent plant, the patterns of resource distribution are determined by vascular continuity and phyllotaxy^9^,^10^,^11^,^12^,^13^. Tracer studies have also found that the efficiency of resource mobilization differed between basipetal and acropetal directions^14^,^15^. The apical and basal stolon internodes seem to differ in the function of carbohydrate supply. Studies found that all growth measures of alligator weed were greater when the ramet emerged from the basal node (with a distal internode) than when it emerged from the apical node (with a proximal internode)^8^. When two nodes are attached with the same internode in one fragment, will the resources in the internode improve the growth of the ramet that emerges from the basal node?

After fragmentation, the burial orientation in soils may be random. Departure from the natural position makes endogenous growth substances reorient due to gravity stimulation^16^,^17^. For example, fragments of horizontal stems of giant reed (*Arundo donax*) had a higher sprouting ability than those of vertical stems when both types of fragments were buried horizontally^18^,^19^. Burial orientation was shown to affect the establishment of small, single-node clonal fragments of alligator weed, and horizontal orientation was better^20^. For fragments with two nodes, are the survival and final size of ramets still the greatest when fragments are buried in the horizontal position? When the adjacent mode of nodes and internodes differs, the distribution of endogenous growth substances (e.g., auxins) in the fragment might change. Therefore, we investigated whether the burial orientation affects their survival and growth depending upon the node and internode adjacent mode. It is possible that fragments with different node and internode adjacent mode are buried in several orientations in nature.

When single-node fragments with internodes are buried in the vertical orientation, there are 3 possible positions of node with respect to the soil surface: node on the soil surface, node under the soil surface, and node above the soil surface (internode inserted in the soil). The position of the node with respect to the soil surface may greatly affect the sprouting of ramet and root because both biotic (e.g., pathogen activities) and abiotic conditions (e.g., nutrient, temperature and moisture) are changed around the node between when the node is above the soil and when it is under the soil surface^21^,^22^,^23^. The sprouting and growth of apical and basal nodes in two-node fragments may be different from those of one-node fragments. The effect of the node being positioned above the soil surface on the survival and growth of alligator weed has not been investigated.

In this study, we conducted two experiments in a phytotron to investigate whether the adjacent mode of the node and internode, fragment type, burial orientation and the position of the node with respect to the soil surface would affect the sprouting, rooting and growth of the stoloniferous, invasive herb alligator weed. In one experiment, we examined stolon fragments differing in the adjacent mode of the node and internode (middle node, apical node and basal node), fragment type (one-node and two-node) and burial orientation (horizontal, upward and downward). In this experiment, the nodes were on the soil surface. In a second experiment, we set the apical node or basal node (in one-node and two-node fragments) on the soil surface, above the soil surface or under the soil surface in a vertical (upward or downward) orientation. We tested the following hypotheses: (1) the adjacent mode of the node and internode will affect the sprouting and growth of two types of fragments, and the growth may be larger in the basal node; (2) burial orientation will alter the effects of the adjacent mode of the node and internode on sprouting and growth; and (3) the position of the node above the soil surface and under the soil surface will decrease the sprouting and growth of alligator weed.

## Results

### Effects of the adjacent mode of the node and internode, fragment type and burial orientation

**Figure 1:**
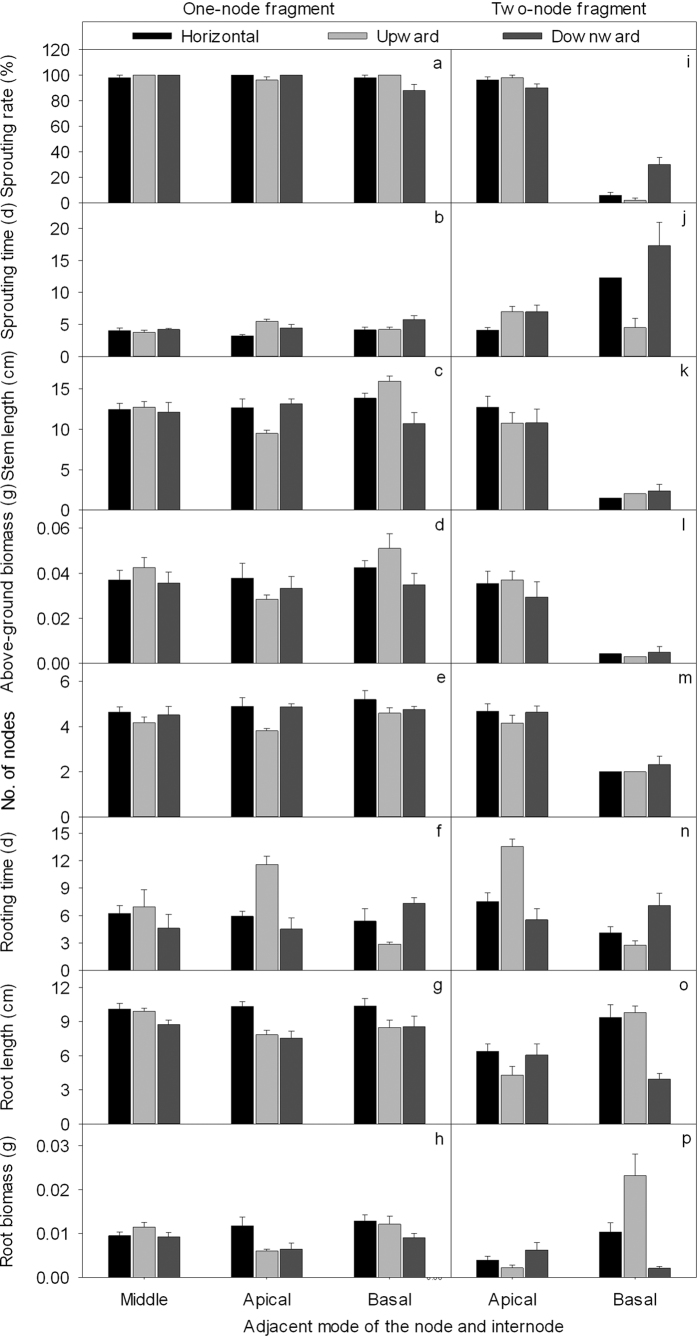
Effects of the adjacent mode of the node and internode (middle, apical, basal), fragment type (one-node, two-node) and burial orientation (horizontal, upward, downward) on sprouting rate (**a,i**), sprouting time (**b,j**), stem length (**c,k**), above-ground biomass (**d,l**), number of nodes (**e,m**), rooting time (**f,n**), root length (**g,o**) and root biomass (**h,p**) of alligator weed. The mean + SE are given.

The adjacent mode of the node and internode significantly affected the sprouting, above-ground growth and root growth of alligator weed (Table 1). The sprouting rate was significantly smaller in basal nodes than those in apical and middle nodes (*P* < 0.05). The sprouting time was significantly longer in basal nodes than those in middle and apical nodes (*P* < 0.05). The rooting time was significantly longer in apical nodes than those in middle nodes and basal nodes (*P* < 0.05). The root length and root biomass were significantly larger in middle nodes and basal nodes than those in apical nodes (Table 1, Fig. 1).

The fragment type significantly affected the sprouting rate, above-ground growth and root length of alligator weed (Table 1). The sprouting rate, stem length, above-ground biomass, node number and root length were significantly larger in one-node than in two-node fragments (*P* < 0.05). The sprouting time was significantly shorter in one-node than in two-node fragments (*P* < 0.05) (Table 1, Fig. 1).

Burial orientation had significant effects on the sprouting time, rooting time, root length and root biomass (Table 1). The sprouting time was significantly longer in the downward orientation than in the horizontal and upward orientations (*P* < 0.05). The rooting time was significantly longer in the upward orientation than in the horizontal and downward orientations (*P* < 0.05). The root length and root biomass were significantly smaller in the downward orientation than in the horizontal and upward orientations (*P* < 0.05) (Table 1, Fig. 1).

The interactive effect of the adjacent mode of the node and internode and fragment type on sprouting, above-ground growth and root growth was significant (Table 1, M × T). For one-node fragments, the basal node increased the stem length, above-ground biomass and node number compared to the middle node and apical node (*P* < 0.05). For two-node fragments, the basal node decreased the sprouting rate, stem length, above-ground biomass and node number compared to the apical node (*P* < 0.05) (Table 1, M × T, Fig. 1).

The interactive effect of the adjacent mode of the node and internode and burial orientation on the sprouting rate, sprouting time, rooting time, root length and root biomass was significant (Table 1, M × O). Irrespective of fragment type, the basal nodes prolonged the sprouting time in the downward orientation compared to horizontal and upward orientations (*P* < 0.05), but this was not the case for the middle node or apical node in the downward orientation (*P* < 0.05). The apical nodes prolonged the rooting time in the upward orientation compared to horizontal and downward orientations (*P* < 0.05), while the basal nodes exhibited the opposite trend (*P* < 0.05), and the difference was not significant between fragments with middle nodes (*P* > 0.05). The apical nodes decreased the root length and root biomass in the upward orientation compared to horizontal and downward orientations (*P* < 0.05), while the basal nodes decreased the root length and root biomass in the downward orientation compared to horizontal and upward orientations (*P* < 0.05). The difference was not significant between fragments with middle nodes (*P* > 0.05) (Table 1, M × O, Fig. 1).

The interactive effect of fragment type and burial orientation on the sprouting rate, sprouting time and root biomass was significant (Table 1, T × O). For the two-node fragments, the downward orientation prolonged the sprouting time, increased the sprouting rate, and decreased the root biomass compared to horizontal and upward orientations (*P* < 0.05), but this was not the case for one-node fragments in the downward orientation (Table 1, T × O, Fig. 1).

Moreover, there were interactive effects of M × O × T on the sprouting rate, sprouting time, stem length, root length and root biomass of alligator weed (Table 1, M × O × T). For one-node fragments, the downward orientation greatly decreased the sprouting rate compared to horizontal and upward orientations in the basal node fragments (*P* < 0.05) but not for middle nodes and apical nodes (Fig. 1a). For two-node fragments, the downward orientation significantly increased the sprouting rate compared to other orientations in basal nodes (*P* < 0.05) but not for apical nodes (Table 1, M × O × T, Fig. 1i). For one-node fragments, the downward orientation prolonged the sprouting time in basal nodes, while the upward orientation prolonged the sprouting time in the apical nodes. For two-node fragments, the upward orientation shortened the sprouting time in the basal nodes (Table 1, M × O × T, Fig. 1b,j). Upward orientation greatly increased the root biomass in the basal nodes of two-node fragments but not in the other treatments (Table 1, M × O × T, Fig. 1h,p).

### Effects of node position with respect to the soil surface and fragment type

**Figure 2:**
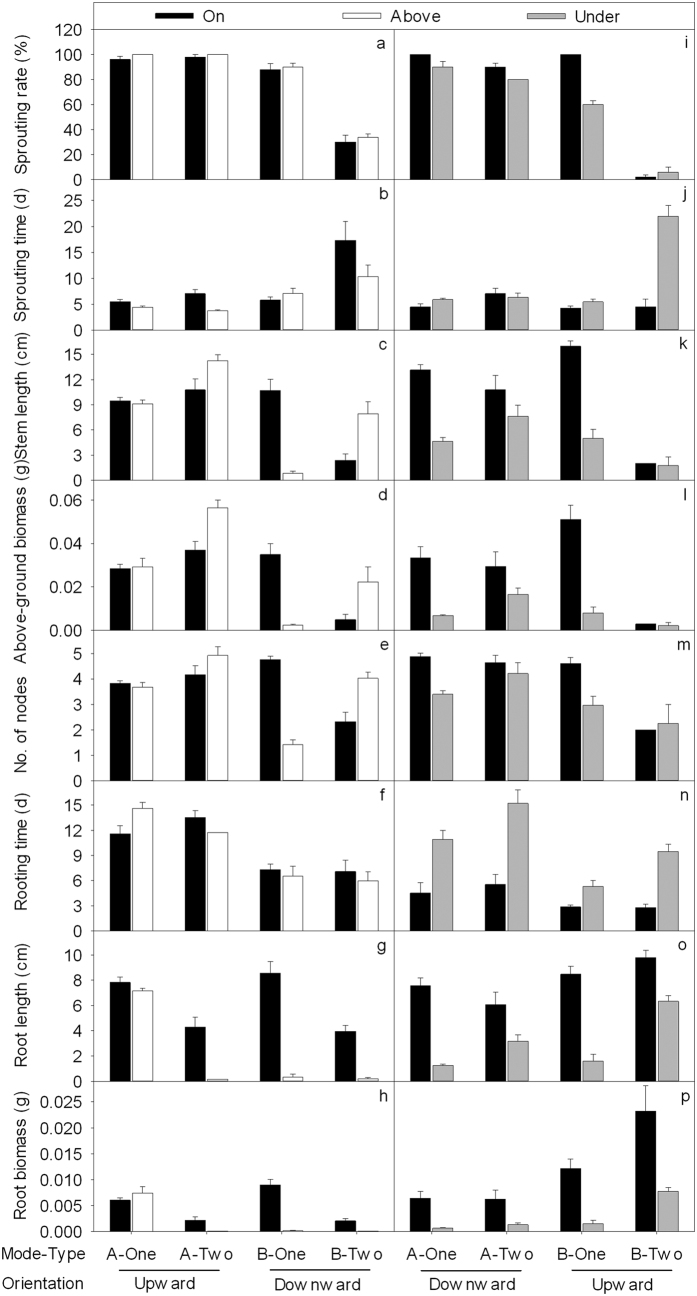
Effects of fragment type (one-node, two-node) and position of the node with respect to the soil surface (on, above, under the soil surface) on sprouting rate (**a,i**), sprouting time (**b,j**), stem length (**c,k**), above-ground biomass (**d,l**), number of nodes (**e,m**), rooting time (**f,n**), root length (**g,o**) and root biomass (**h,p**) of apical node (A) or basal node (B) alligator weed fragments buried upward or downward. The mean + SE are given.

In the treatments of fragments with the apical node buried upward (on the soil surface and above the soil surface) (Fig. 2a–h: A-One/Two – Upward), the fragment type significantly affected the above-ground and root growth (Table 2). Stem length, above-ground biomass and node number were significantly larger (*P* < 0.05) (Fig. 2c–e) and the root length and root biomass were significantly smaller in the two-node fragments than those in the one-node fragments (*P* < 0.05) (Fig. 2g,h). The position of node with respect to the soil surface had significant effects on the sprouting time, above-ground biomass and root length (Table 2). In the node above the soil surface, the sprouting time was shorter, the above-ground biomass was larger and the root length was smaller than those in the node on the soil surface (*P* < 0.05) (Fig. 2a,b,d,g). The interactive effect of fragment type and position of the node with respect to the soil surface was significant (Table 2). For two-node fragments, the node above the soil surface significantly increased the stem length and above-ground biomass and decreased the root length compared to those nodes on the soil surface, but this was not the case for one-node fragments (*P* < 0.05) (Fig. 2c,d,g).

In the treatments of fragments with the basal nodes buried downward (on the soil surface and above the soil surface) (Fig. 2a–h: B-One/Two – Downward), the fragment type significantly affected the sprouting rate, sprouting time, root length and root biomass (Table 2). The sprouting time was significantly longer and the sprouting rate, root length and root biomass were significantly smaller in two-node fragments than in one-node fragments (*P* < 0.05) (Fig. 2a,b,g,h). Position of the node with respect to the soil surface had significant effects on the node number, root growth (Table 2), node number, root length and root biomass, which were significantly smaller in the nodes above the soil surface than in the nodes on the soil surface (*P* < 0.05) (Fig. 2e,g,h). The interactive effect of fragment type and position of the node with respect to the soil surface was significant (Table 2). For two-node fragments, the nodes above the soil surface significantly increased the stem length, above-ground biomass and node number compared to those nodes on the soil surface, but this was not the case for one-node fragments (*P* < 0.05) (Fig. 2c–e).

In the treatments of fragments with apical nodes buried downward (on the soil surface and under the soil surface) (Fig. 2i–p: A-One/Two – Downward), the fragment type significantly affected the sprouting rate (Table 2). The sprouting rate was smaller in two-node fragments than in one-node fragments (*P* < 0.05) (Fig. 2i). The position of the node with respect to the soil surface had significant effects on almost all of the measures (Table 2). The sprouting rate, stem length, above-ground biomass, node number, root length, root biomass were significantly smaller and the rooting time was longer in nodes under the soil surface than nodes on the soil surface (*P* < 0.05) (Fig. 2i,k–p). The interactive effect of fragment type and position of the node with respect to the soil surface was significant on stem length and root length (Table 2). Nodes under the soil surface decreased the stem length and root length more in one-node fragments than in two-node fragments (*P* < 0.05) (Fig. 2k,o).

In the treatments of fragments with basal nodes buried upward (on the soil surface and under the soil surface) (Fig. 2i–p: B-One/Two – Upward), the fragment type significantly affected all of the measurements (Table 2). The sprouting rate, stem length, above-ground biomass and node number were significantly smaller; the root length and root biomass were larger; and the sprouting time and rooting time were longer in two-node fragments than in one-node fragments (*P* < 0.05) (Fig. 2i–p). The position of the node with respect to the soil surface had significant effects on nearly all of the measures (Table 2). The sprouting rate, stem length, above-ground biomass, root length and root biomass were significantly smaller and the sprouting time and rooting time were longer in nodes under the soil surface than those nodes on the soil surface (*P* < 0.05) (Fig. 2i–l,n–p). The interactive effect of fragment type and position of node and soil surface was significant (Table 2). Nodes under the soil surface decreased the sprouting rate, stem length, above-ground biomass and root length more in one-node fragments than in two-node fragments (*P* < 0.05) (Fig. 2i,k,l,o), and prolonged sprouting time and rooting time greater in two-node fragments than in one-node fragments (*P* < 0.05) (Fig. 2j,n).

## Discussion

Basal nodes decreased the sprouting rate but improved the root growth of both one-node and two-node fragments. Basal nodes improved the above-ground growth of one-node fragments but not two-node fragments. These results suggested that the effect of the adjacent mode of the node and internode differed on growth in the two fragment types. Burial orientation altered the effects of the adjacent mode of the node and internode on the sprouting rate, sprouting time, root length and root biomass. Thus, the results partly supported our first two hypotheses, suggesting that the effects of the adjacent mode of the node and internode on the sprouting and growth of small fragments depend on fragment type, and burial orientation changed the interactive effects of fragment type and the adjacent mode of the node and internode on alligator weed.

Previous studies showed that attached stolon internodes substantially increased the probability of survival of severed, juvenile ramets^7^. The carbohydrates in stem and rhizome are important for growth after resting periods and after herbivory or other damage to active plant structures^7^,^24^,^25^,^26^. There was significant growth from both the basal and apical nodes depending on the orientation and fragment type in our research. These results suggested that the carbohydrate and nitrogen reserves in the internode may be mobilized to both the basal node and apical node. However, evidence showed that at low temperature, the survival rate of the *Eremochloa ophiuroides* stolons was positively correlated with the carbohydrate content in stolon internodes^27^. Hence, carbohydrate support from stolon internodes was perhaps important only at the beginning of the regrowth of the plant fragments^28^. Therefore, it may be that only the sprouting rate of alligator weed fragments is affected by the carbohydrate and nitrogen stored in internodes. In a previous experiment, the survival rate was significantly lower in basal nodes than in apical and middle nodes^8^. Therefore, we speculate that the sprouting rate of ramets from nodes may be more affected by reserves in basal internodes than in apical internodes for fragments with only one node. The basal internode may mobilize more carbohydrates and soluble proteins to ramets to sprout in one-node fragments. Previous studies showed that the survival rate was 46% for the fragments without stolon internodes^8^, which suggested that fragments of alligator weed could sprout by using fewer reserves stored in the node. In this study, the stolon fragments used for the experiments were thick; thus, the reserves in nodes may have been sufficient for sprouting and rooting^29^. Hence, the adjacent mode of the node and internode did not affect the sprouting rate of basal nodes in horizontal and upward orientations in one-node fragments in our experiment.

The sprouting and rooting processes of fragments undergo complex regulation by multiple hormones, i.e., auxin and cytokinins^30^. Auxin distributes mainly in nodes and transports towards the base of the stem. Auxins were reduced in the apical node and accumulated in the basal node. Reduced auxin in the apical node promoted sprouting of stems, and increased auxin in basal nodes promoted rooting. Therefore, basal nodes improved root biomass and shortened rooting time compared to apical nodes. Numerous roots assimilate more water and nutrients and then promote above-ground growth^31^. The node in a one-node fragment will both sprout and root. For a two-node fragment, apical nodes mainly sprout more and root less and basal nodes mainly root more and sprout less. This was the reason the basal node of the two-node fragment (Fig. 1) exhibited low above-ground growth. The fragment type significantly affected the above-ground and root growth measures of alligator weed.

Burial orientation was important in the rooting process of one-node and two-node fragments. Hormonal transportation involves geotropism in the stem. When the stem was upward, the hormones in the apical node were transported more to the basal node than when the stem was in horizontal and downward orientations. In two-node fragments buried facing upward, the basal node had the hormones from the apical node transported to it and the hormones from itself, resulting the largest final root biomass and shortest rooting time (Fig. 1, Two-node fragment – Basal – Upward). The upward orientation caused the apical node to lose more hormones, resulting in the smallest final root biomass and longest rooting time. The results indicated that the upward, horizontal orientation improved the root development in the basal node of the two-node stolon fragments. The rooting process was affected by many other conditions, such as storage utilization and moisture. The downward orientation decreased the root biomass of stolon fragments, and this orientation may have decreased the utilization of reserves among these fragments^8^,^30^.

In the second half of our experiment, the apical node buried upward and the basal node buried downward of two-node fragments exhibited improved stem length and above-ground biomass in nodes above the soil surface compared to those in nodes on the soil surface. The other treatments decreased the sprouting and growth in nodes above the soil surface and under the soil surface compared to those in nodes on the soil surface. Thus, our results partially supported the third hypothesis, suggesting that not all of the positions of nodes above and under the soil surface decreased the sprouting and growth of alligator weed fragments.

In the treatment with apical nodes buried upward for one-node fragments, although the nodes did not touch the soil, ramets sprouting from apical nodes could grow because the attached basal internodes were able to root. However, the rooting time was long; thus, the root length was short. For two-node fragments, ramets from apical nodes grew well although the apical nodes above the soil surface had small roots, which because basal nodes in the same fragment were on the soil surface (Fig. 2 B-Two –Upward – On the soil surface) and could root to absorb water and nutrients to supply the ramets of apical nodes. Our experiment may provide the first published suggestion that when a node is above the soil surface on a one-node fragment buried upward, ramets can survive and grow and the basal internode can root. This phenomenon greatly increased the survival rate of the fragment that broke off from mother plant and fell to the ground, but the node did not reach the soil. This effect should be considered during the management and control of alligator weed.

In the treatment with basal nodes buried downward, for one-node fragments, when the node was above the soil surface and the attached apical internode was inserted into the soil, the apical internode could not root. In addition, the roots from the node could not touch the soil and could not absorb water; thus, the stem of the ramet and root were small and even died. Of the 44 sprouted ramets with basal nodes above the soil surface in one-node fragments buried downward, 31.82% of fragments’ apical internodes rotted ten days after planting. The basal node broke off from apical internode and fell to the soil surface; then, the root from the basal node could absorb water, enabling the ramet to grow. The ramets of the other 68.18% of fragments that attached at the apical internode did not rot; they may have died if the plants had not been harvested after one month of planting. The survival of ramets mainly depended on the ability of ramets to produce roots and take up water^7^. When the node was above the soil surface and the apical internode or basal internode was inserted into the soil, only the basal internode could produce roots, giving evidence of hormonal polarity transport in the stem.

The treatment under the soil surface decreased the sprouting rate, above-ground growth and root growth, regardless of whether the fragment was one-node or two-node, apical node or basal node, or buried upward or downward. The basal node of a one-node fragment buried upward under the soil surface mainly decreased the survival rate. Although we showed that the sprouting rate of basal nodes of two-node fragments buried upward (Fig. 2i) was low, the apical nodes in the same two-node fragments (Fig. 2 B-Two – Upward – On the soil surface and Fig. 2 A-Two – Upward – Above the soil surface were at the same fragment; Fig. 2 B-Two – Upward – Under the soil surface and Fig. 2 A-Two – Upward – On the soil surface were at the same fragment) exhibited high survival rates and above-ground growth. Therefore, as the whole fragment of a two-node fragment, the effect of buried under soil surface was smaller than in the one-node fragment. The node under the soil surface could decrease the vegetative propagation of alligator weed. This result was consistent with previous studies of alligator weed^32^ and similar to published studies of other herbs^22^,^23^.

Alligator weed has strong vegetative propagation ability, like many other invasive plants, such as *Solidago canadensis*^33^. This is one reason why some plant species are more invasive^34^. Disturbance often occurs in natural habitats, and alligator weed stems are fragile and easy severed with the interference of humans, animals, floods, and other factors. The survival rate was very high in many treatments in our experiment; thus, fragments that are cut into a single node could propagate regardless of the adjacent mode of the node and internode. If the fragmentation occurred, regardless of whether fragments’ nodes touched the soil, were buried into the soil or were on the surface, most fragments sprouted new stems and established a new population, which was then invasive in a new place.

In all of the treatments, optimal growth was observed in ramets that sprouted from fragments with basal nodes (including one-node and two-node fragments) buried upward on the soil surface. These treatments might be advantageous for use in clonal propagation. As a whole fragment, one-node fragments were more easily affected by environmental conditions than two-node fragments, such as the basal node of a one-node fragment oriented downward above the soil surface and the basal node of a one-node fragment buried upward under the soil surface. Therefore, reducing the number of nodes of fragments and burying them under or facing downward above the soil surface might be effective methods of controlling the invasion of alligator weed in interferential environment.

## Materials and Methods

### Ethics statement

The experimental plants were obtained from the greenhouse of Northeast Normal University, Jilin Province, China, and no permission was required for the collection. The experimental conditions were established by the research team, and no special permission was required for the experiment. The experiment did not involve any endangered or protected species; therefore, no specific permissions were required to collect plants from this location.

### Species

Alligator weed (*Alternanthera philoxeroides* (Martius) Griseb.) is an invasive wetland herb originating from the Parana River region of South America^35^,^36^. It is an invasive weed that has encroached onto a wide range of habitats in southern China and other regions of the world, including the USA, Australia and other parts of Asia. Alligator weed is a stoloniferous and rhizomatous perennial that rapidly grows in both terrestrial and aquatic habitats^37^. The node in the stolon can root in moist conditions, and the axillary bud can develop into a new stolon and ramet or inflorescence. This species does not sexually reproduce in its introduced range. Its primary means of dispersal is via stolons connected to an established population or through stem fragmentation, where stem fragments break off, lodging elsewhere and creating new infestations^5^,^38^.

### Experimental design

**Figure 3:**
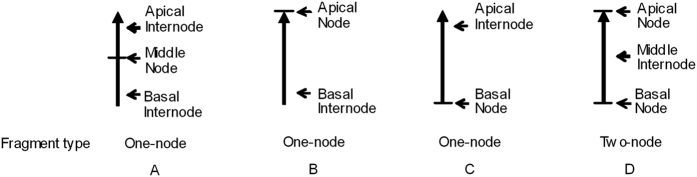
Schematic illustration of the experimental stolon fragments.

Alligator weed plants were collected from Guizhou Province in southwestern China in early April 2015 and vegetatively propagated for more than 4 months in a greenhouse at Northeast Normal University, Jilin Province, northeastern China. In late August 2015, 1400 clonal fragments of similar diameter were severed from the stock plants of this population for use in the experiment. Each fragment was 4 cm long and derived from the mature, creeping stems of alligator weed. There were three levels of the adjacent mode of the node and internode, including middle node (Fig. 3A), apical node (Fig. 3B,D) and basal node (Fig. 3C,D). If the fragment internode consisted of both apical and basal internodes, each included half of the total length. There were two levels of fragment types: one-node (Fig. 3A,B and C) and two-node (Fig. 3D). Each two-node fragment (two end nodes) contained an apical node, a basal node and the middle internode. Each one-node fragment contained a node and an internode or two internodes. Of the 1400 stolon fragments, 900 were selected and used for the experiments and 40 were used for the initial measurements.

The experiments started on 23 August 2015 and ended 4 weeks later on 24 September. The experiments were conducted in a phytotron at the Key Laboratory of Vegetation Ecology of Northeast Normal University. During the experiments, the relative air humidity was 55%, the light:dark cycle was 12:12 h and the day:night temperature cycle was 25 °C:20 °C. At the start of the experiments, the average diameter of the stolon fragments was 0.33 ± 0.03 cm (mean ± SD, N = 10). The dry weights of the middle node, apical node, basal node of one-node fragments and two-node fragments were 0.0205 ± 0.0081 g, 0.0208 ± 0.0070 g, 0.0200 ± 0.0065 g, 0.0191 ± 0.0066 g (mean ± SD, N = 10), respectively.

**Figure 4:**
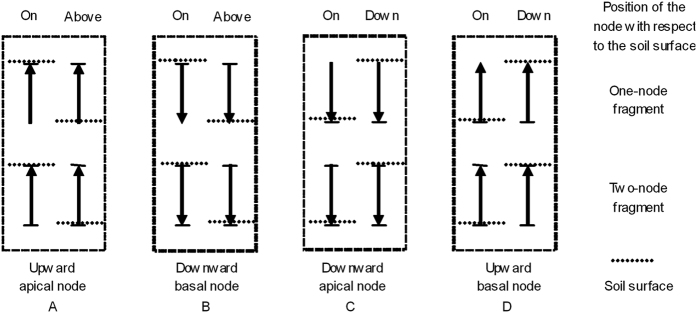
Schematic illustration of the position of the node with respect to the soil surface.

The study consisted of two experiments. In the first experiment, the aim was to investigate the effects and interactive effects of node adjacent mode, fragment type and burial orientation on the sprouting and growth of alligator weed. There were three levels of the adjacent mode of the node and internode (middle node, apical node and basal node), combined with two levels of fragment type (one-node and two-node) and three levels of burial orientation (horizontal, upward and downward). In this experiment, the nodes of one-node fragments and the compared nodes of two-node fragments were buried on the soil surface and measured. In the second experiment, the aim was to investigate the effects and interactive effects of fragment type and position of the node with respect to the soil surface on the sprouting and growth of alligator weed. There were two levels of fragment type (one-node and two-node) combined with the position of the node with respect to the soil surface (on, above or under the soil surface) (Fig. 4). The burial orientations were upward and downward. In this experiment, the fragments with apical nodes (including one-node and two-node) buried upward were one group and were divided into two positions of node with respect to the soil surface (apical node on the soil surface and above the soil surface) (Fig. 4A). The fragments with basal nodes buried downward were one group and were divided into two positions of node with respect to the soil surface (basal node on the soil surface and above the soil surface) (Fig. 4B). The fragments with apical nodes buried downward were one group and were divided into two positions of node with respect to the soil surface (apical node on the soil surface and under the soil surface at 4 cm depth) (Fig. 4C). The fragments with basal nodes buried upward were one group and were divided into two positions of node with respect to the soil surface (basal node on the soil surface and under the soil surface at 4 cm depth) (Fig. 4D). The treatments of nodes on the soil surface were shared by the two experiments.

In the two experiments, 90 plastic pots (18 cm × 13 cm × 7 cm; length × width × height) were used. Each pot was planted with 10 stolon fragments. The substrate used in the pot was sand. Each pot was fertilized with 15 mL of Hoagland nutrient solution once every two weeks. Tap water was supplied once per day to keep the soil moist. We checked the sprouting and rooting status daily until the node sprouted and rooted. For the node buried below the soil surface, we pushed aside the soil and observed the node then covered the soil again. During the experiments, the pots were systematically repositioned to avoid the effects of possible environmental patchiness within the phytotron. There were five replicates of boxes for each of the 18 treatments in the two experiments.

### Measurements and analyses

On 24 September, we counted the number of nodes and measured the stem length of each stem and the longest root length that had rooted from the stolon fragment. We calculated the percentage of sprouting rate and the mean values of size of the survived fragments in each pot. Then, we divided each ramet into roots, stems and leaves. All of the parts were then dried at 75 °C for 72 h and weighed. The sprouting rate is ratio of nodes that sprout stem to total nodes of a given type. The sprouting time only included the nodes that exhibited sprouting during the experiment. The stem length and number of nodes were the sum of these values in all of the stems sprouted from one node. The above-ground biomass was the sum of all leaves and stems sprouted from one node. The old stolon fragments were not used in the biomass analyses.

For the first experiment, a three-way ANOVA was conducted to test the effects of the adjacent mode of the node and internode, fragment type and burial orientation on the sprouting rate, sprouting time, rooting time, stem length, above-ground biomass, number of nodes, root length and root biomass of alligator weed. For the second experiment, a two-way ANOVA was conducted to test the effects of fragment type and position of node and soil surface on the measures mentioned above. If significant effects were detected, then Tukey’s multiple comparison tests were used to compare the mean values between the treatments. Prior to the ANOVAs, all of the data were checked for normality and homoscedasticity. All of the statistical analyses were carried out with SPSS 20.0 software (Chicago, IL, USA). The differences were considered to be significant if *P* < 0.05.

## Additional Information

**How to cite this article:** Peng, X. *et al*. Vegetative propagation capacity of invasive alligator weed through small stolon fragments under different treatments. *Sci. Rep.* **7**, 43826; doi: 10.1038/srep43826 (2017).

**Publisher's note:** Springer Nature remains neutral with regard to jurisdictional claims in published maps and institutional affiliations.

## Figures and Tables

**Table 1:** ANOVAs for effects of the adjacent mode of the node and internode (middle node, apical node and basal node), fragment type (one-node and two-node) and burial orientation (horizontal, upward and downward), and their interactions on sprouting and growth of the final size of alligator weed.

	DF	Sprouting rate	Sprouting time	Stem length	Above-ground biomass	No. of nodes	Rooting time	Root length	Root biomass
Adjacent mode (M)	2	418.79***	9.12***	12.31***	3.77*	12.10***	13.67***	7.24**	14.32***
Fragment type (T)	1	862.24***	38.51***	58.02***	26.57***	41.36***	0.67 ns	32.77***	2.78 ns
Burial orientation (O)	2	2.66^#^	11.19***	0.35 ns	0.79 ns	2.22 ns	4.40*	14.05***	8.65***
M × T	1	710.41***	13.78***	51.74***	28.75***	39.01***	2.91^#^	4.02*	4.77*
M × O	4	3.18*	7.38***	1.58 ns	0.30 ns	0.45 ns	16.70***	4.44**	9.21***
T × O	2	10.00***	8.48***	0.18 ns	0.10 ns	0.52 ns	0.15 ns	2.20 ns	6.81**
M × T × O	2	28.37***	6.28**	3.69*	1.57 ns	0.43 ns	0.15 ns	9.67***	9.24***

Degrees of freedom (DF), F values and the significance levels (***P < 0.001, **P < 0.01, *P < 0.05, ^#^*P* < 0.1, ns *P* ≥ 0.1) are given.

**Table 2:** ANOVAs for the effects of fragment type (one-node and two-node) and position of the node with respect to the soil surface (on, above and under the soil surface) on sprouting and growth of the final size of alligator weed.

		DF	Sprouting rate	Sprouting time	Stem length	Above-ground biomass	No. of nodes	Rooting time	Root length	Root biomass
Apical node upward (on or above the soil surface)	Type	1	0.40 ns	0.99 ns	16.36***	26.75***	8.72**	0.15 ns	49.88***	22.79***
	Position	1	3.60^#^	22.55***	3.73^#^	8.45*	1.29 ns	0.27 ns	10.46**	0.08 ns
	T × P	1	0.40 ns	5.43*	5.83*	7.35*	2.74 ns	4.19^#^	5.30*	2.13 ns
Basal node downward (on or above the soil surface)	Type	1	185.66***	13.05**	0.36 ns	1.57 ns	0.12 ns	0.13 ns	11.82**	22.2***
	Position	1	0.51 ns	1.97 ns	4.27^#^	3.45^#^	11.60**	0.77 ns	75.35***	53.27***
	T × P	1	0.06 ns	4.20^#^	53.61***	37.47***	109.86***	0.02 ns	10.54**	21.29***
Apical node downward (on or under the soil surface)	Type	1	13.33**	4.28^#^	0.07 ns	0.42 ns	1.08 ns	4.13^#^	0.11 ns	0.05 ns
	Position	1	13.33**	0.27 ns	26.05***	18.79***	12.37**	37.94***	51.98***	23.81***
	T × P	1	0 ns	2.12 ns	5.42*	2.26 ns	3.70^#^	1.56 ns	7.05*	0.13 ns
Basal node upward (on or under the soil surface)	Type	1	770.13***	97.14***	41.37***	13.67**	10.99**	11.28**	29.23***	10.50**
	Position	1	43.20***	120.28***	17.47**	9.01*	1.91 ns	56.59***	85.51***	23.90***
	T × P	1	64.53***	90.78***	16.09**	8.37*	3.54^#^	12.18**	9.31**	0.79 ns

Degree of freedom (DF), F values and the significance levels (***P < 0.001, **P < 0.01, *P < 0.05, ^#^*P* < 0.1, ns *P* ≥ 0.1) are given.
